# Tendon grafts with preserved muscle demonstrate similar biomechanical properties to tendon grafts stripped of muscular attachments: a biomechanical evaluation in a porcine model

**DOI:** 10.1186/s40634-021-00375-6

**Published:** 2021-08-02

**Authors:** Luis Fernando Zukanovich Funchal, Diego Costa Astur, André Luiz Almeida Pizzolatti, Arthur Paiva Grimaldi, Andrew Esteban Jimenez, Ari Digiácomo Ocampo Moré, Carlos Rodrigo de Mello Roesler, Moises Cohen

**Affiliations:** 1 Biomechanical Laboratory From Universidade Federal de Santa Catarina, Hospital Baia Sul, Florianópolis, Brazil; 2 Universidade Federal de São Paulo, Hospital Samaritano and Instituto Astur, Av Pacaembu 1024, São Paulo, SP 01234-000 Brazil; 3 Biomechanical Laboratory From Universidade Federal de Santa Catarina, Florianópolis, Brazil; 4 University of Connecticut, Storrs, USA; 5 Laboratório de Engenharia Biomecânica Do Hospital Universitário da UFSC, Florianópolis, Brazil; 6 Department and Chief From Biomechanical Laboratory, Universidade Federal de Santa Catarina, Florianópolis, Brazil; 7 Orthoapedic Surgeon From Universidade Federal de São Paulo, São Paulo, Brazil

**Keywords:** Autograft, Flexor tendon, Graft failure, Uniaxial tension, Biomechanical

## Abstract

**Purpose:**

(1) To evaluate the biomechanical properties of a porcine flexor digitorum superficialis tendon graft with preserved muscle fibers and (2) to compare these results with the biomechanical properties of a porcine tendon graft after removal of associated muscle.

**Methods:**

Eighty-two porcine forelegs were dissected and the flexor digitorum superficialis muscle tendons were harvested. The study comprised of two groups: Group 1 (G1), harvested tendon with preserved muscle tissue; and Group 2 (G2), harvested contralateral tendon with removal of all muscle tissue. Tests in both groups were conducted using an electro-mechanical material testing machine (Instron, model 23-5S, Instron Corp., Canton, MA, USA) with a 500 N force transducer. Yield load, stiffness, and maximum load were evaluated and compared between groups.

**Results:**

The behavior of the autografts during the tests followed the same stretching, deformation, and failure patterns as those observed in human autografts subjected to axial strain. There were no significant differences in the comparison between groups for ultimate load to failure (*p* = 0.105), stiffness (*p* = 0.097), and energy (*p* = 0.761).

**Conclusion:**

In this porcine model biomechanical study, using autograft tendon with preserved muscle showed no statistically significant differences for yield load, stiffness, or maximum load compared to autograft tendon without preserved muscle. The preservation of muscle on the autograft tendon did not compromise the mechanical properties of the autograft.

**Level of evidence:**

Level III Controlled laboratory study

## Introduction

Semitendinosus (ST) and gracilis (G) tendons have been increasingly used as grafts for numerous tendon and ligament structures because of their anatomical features, including a straightforward harvesting technique [6, 8, 11, 25, 27]. These tendons are also commonly used for anterior cruciate ligament (ACL) reconstruction, elbow ligament reconstruction, medial patellofemoral ligament reconstruction, and many other injuries [1, 22, 23, 26, 30–32].

When used in ACL reconstruction, hamstring autografts undergo a ligamentization process [15]. This remodeling process involves recellularization, revascularization, changes in the collagen structure, decreased density of type I collagen fibers, and increased type III collagen ( which has lower mechanical strength than type I collagen) [2, 3, 13, 24]. During the early stages of healing, the graft is not well incorporated into the bone and may be susceptible to retear when excessive loads are applied to the knee [18, 19]. Autograft tendons are commonly stripped of the adjacent muscular tissue before graft preparation [29]. This process inevitably causes a certain degree of trauma to the tendon and, on a cellular level, may compromise graft healing [17]. Funchal et al., described the clinical and histological advantages of preserving the adherent muscle tissue during ACL reconstruction, with improved knee function scores, return to sport, and increased final size of the autograft used [9].

Thus, the aim of this study was (1) to evaluate the biomechanical properties of a porcine flexor digitorum superficialis tendon graft with preserved muscle fibers and (2) to compare these results with the biomechanical properties of a porcine tendon graft after removal of associated muscle. We hypothesized that the preservation of muscle fibers adhered to the harvested tendon would not make it more susceptible to failure.

## Material and methods

A total of eighty-two porcine forelimbs from two MS60 and F1 breeds were evaluated and their flexor digitorum superficialis muscle tendons were harvested for biomechanical analysis. This was a controlled laboratory study with an allocation ratio of 1:1.

The research was approved by the Research Ethics Committee of the institution (Hospital Governador Celso Ramos – 26,821,019.2.0000.5360).

Inclusion criteria were as follows: pigs aged 28 ± 1 weeks, weighing 105 ± 5 kg and without any evident malformation and/or deformities of their limbs. Pigs were then sacrificed by a veterinarian and their forelimbs were dissected bilaterally. In case of anatomical anomaly or failure of flexor tendon removal, the pair of limbs (right and left) were excluded from the study.

**Fig. 1:**
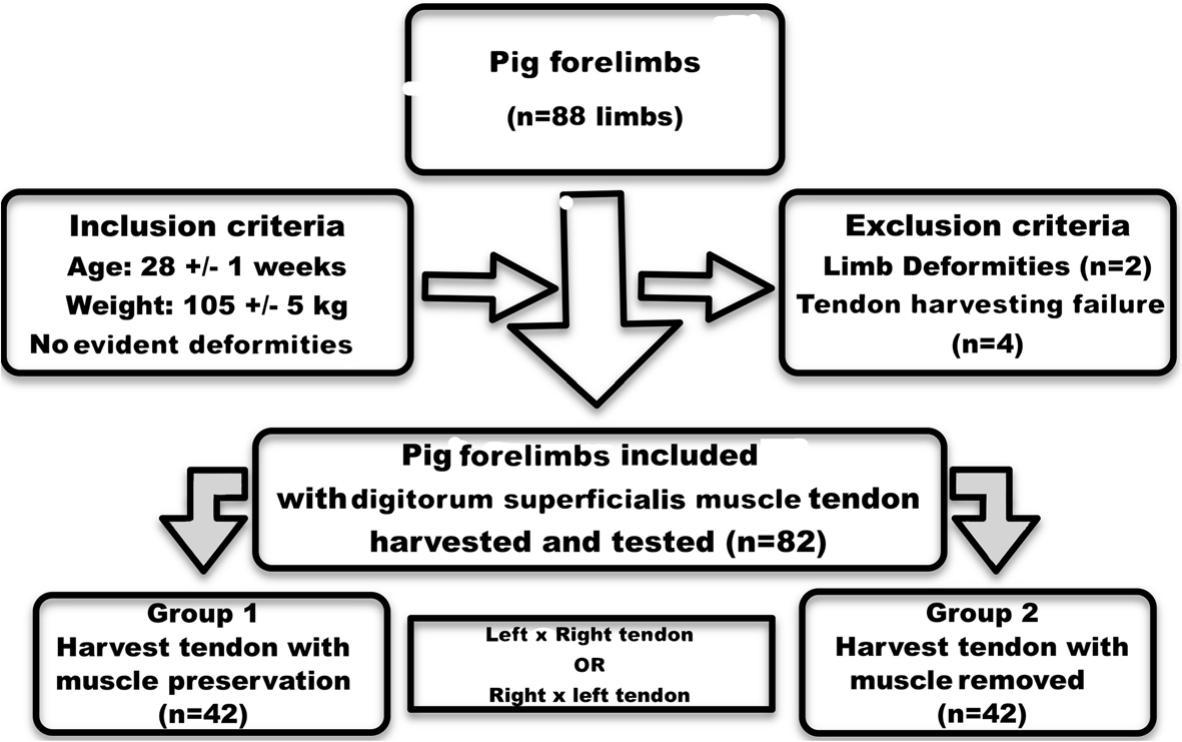
Flowchart for the selection of limbs included in the evaluation. A total of 41 tendons were evaluated in each group

Two groups were randomized using sealed envelopes—Group 1: harvested flexor tendon with muscle preservation; and Group 2: harvested contralateral tendon with removal of all muscle tissue (Fig. 1).

### Flexor tendon harvest technique

**Fig. 2:**
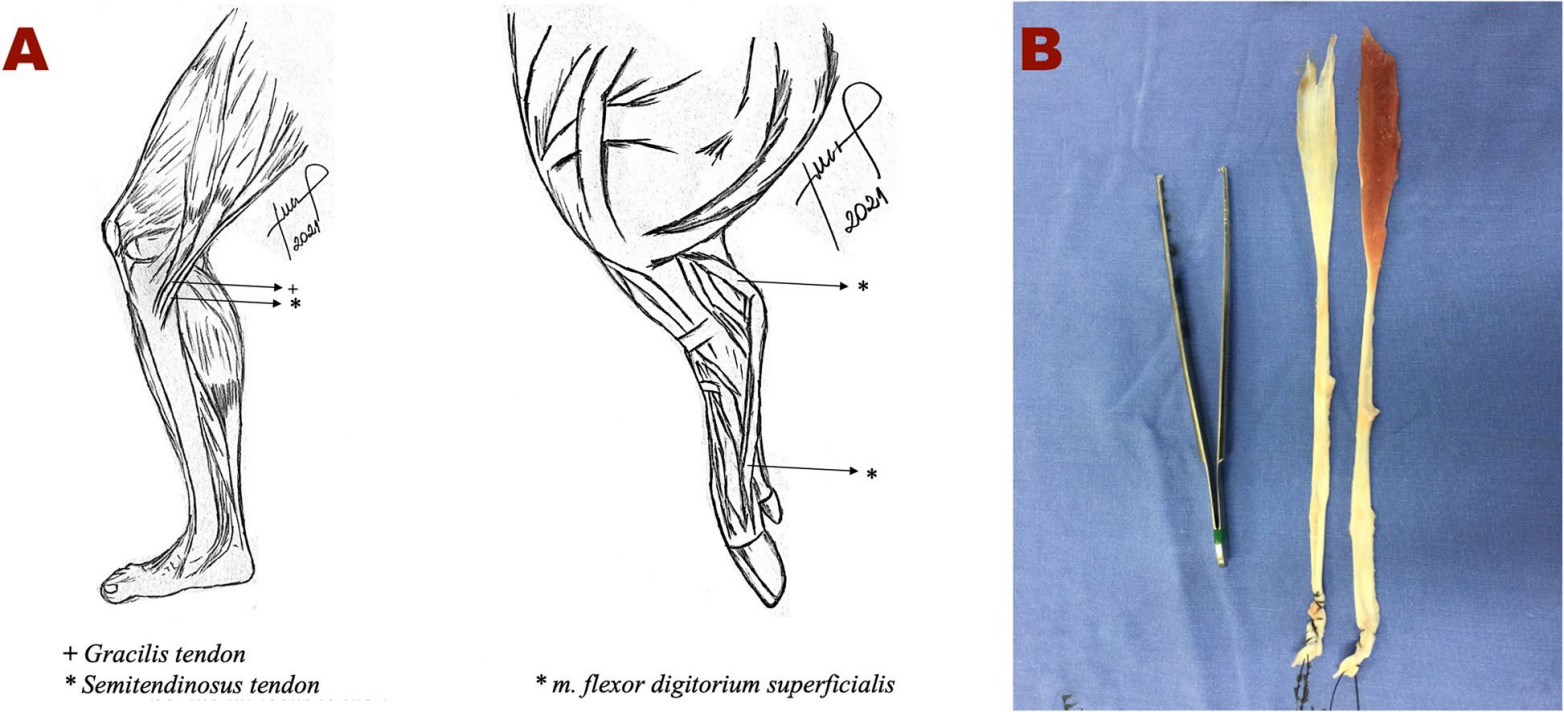
**A** shows the anatomical comparison between the human semitendinosus and gracilis tendons (knee flexors) (left), and the porcine flexor digitorum superficialis (right). The two musculotendinous complexes are quite similar in function and assist with the flexion of the leg at knee. In the biomechanical evaluation of the porcine flexor digitorum superficialis muscle tendon, with and without muscle (**B**), we attempted to identify the most similar structure with the flexor tendons used for ACL reconstruction

The flexor digitorum superficialis tendons of the two forelimbs were harvested from each of the 41 pigs, totaling 82 flexor tendon autografts. The anatomy of the porcine forelimb flexor digitorum tendon is sufficiently similar to that of the human semitendinosus and gracilis tendons (knee flexors) and makes it a suitable alternative for testing purposes [4, 14, 21]. The harvest followed the same technique as hamstring tendon harvest in humans as shown in Fig. 2 [8].

**Fig. 3:**
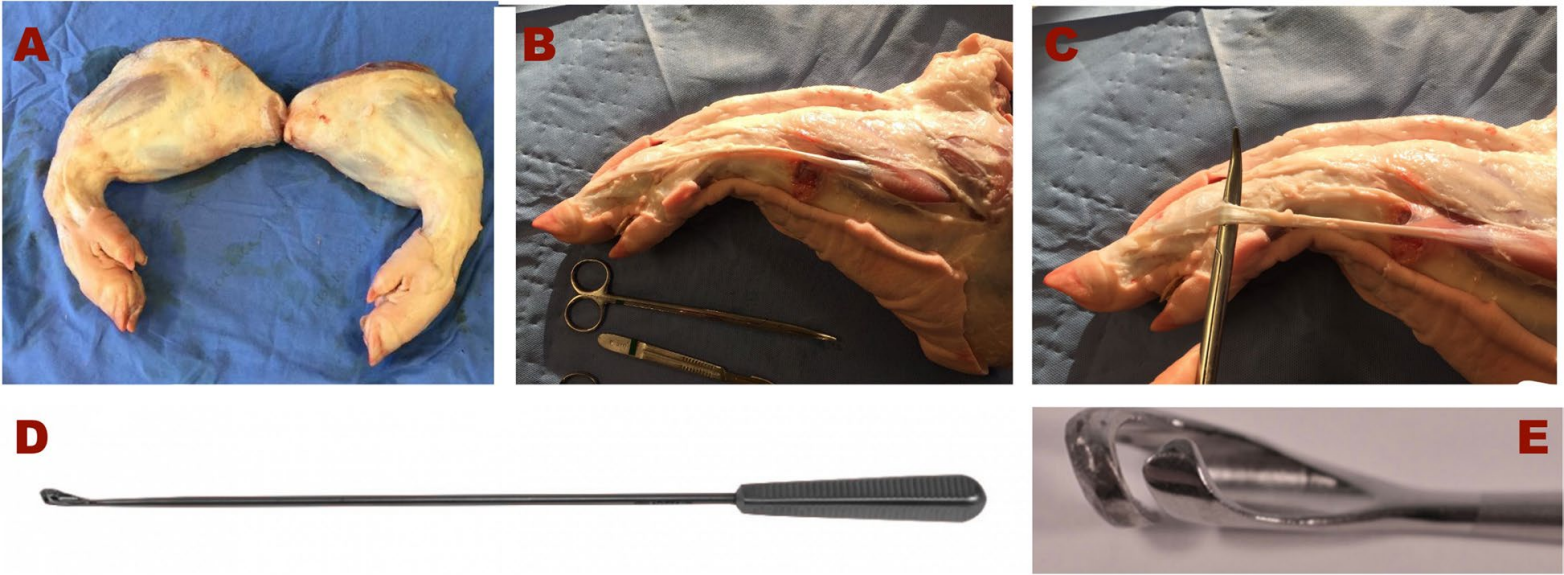
The three stages of dissection of porcine flexor digitorum superficialis tendon. **A** shows the right and left limbs before dissection. **B** and **C** exhibits the pes anserinus after dissection of the superficial layers of the leg. **D** shows a standard tendon stripper (Smith & Nephew/Acufex Slotted Tendon Stripper), and **E**, the details of the open-end of the graft harvester, which is used to release the tendon proximately without distal disinsertion

The flexor digitorum superficialis muscle tendons were identified. Next, the tendons were harvested using tendon strippers (Smith & Nephew/Acufex Slotted Tendon Stripper®), the same instruments used for the harvesting of human hamstring tendons. If the tendon of a limb was selected for G1, all muscular attachments were maintained. Conversely, the tendon of the contralateral limb of the same animal was included in G2, and all muscles adjacent to the tendon were removed (Fig. 3).

### Biomechanical test

**Fig. 4:**
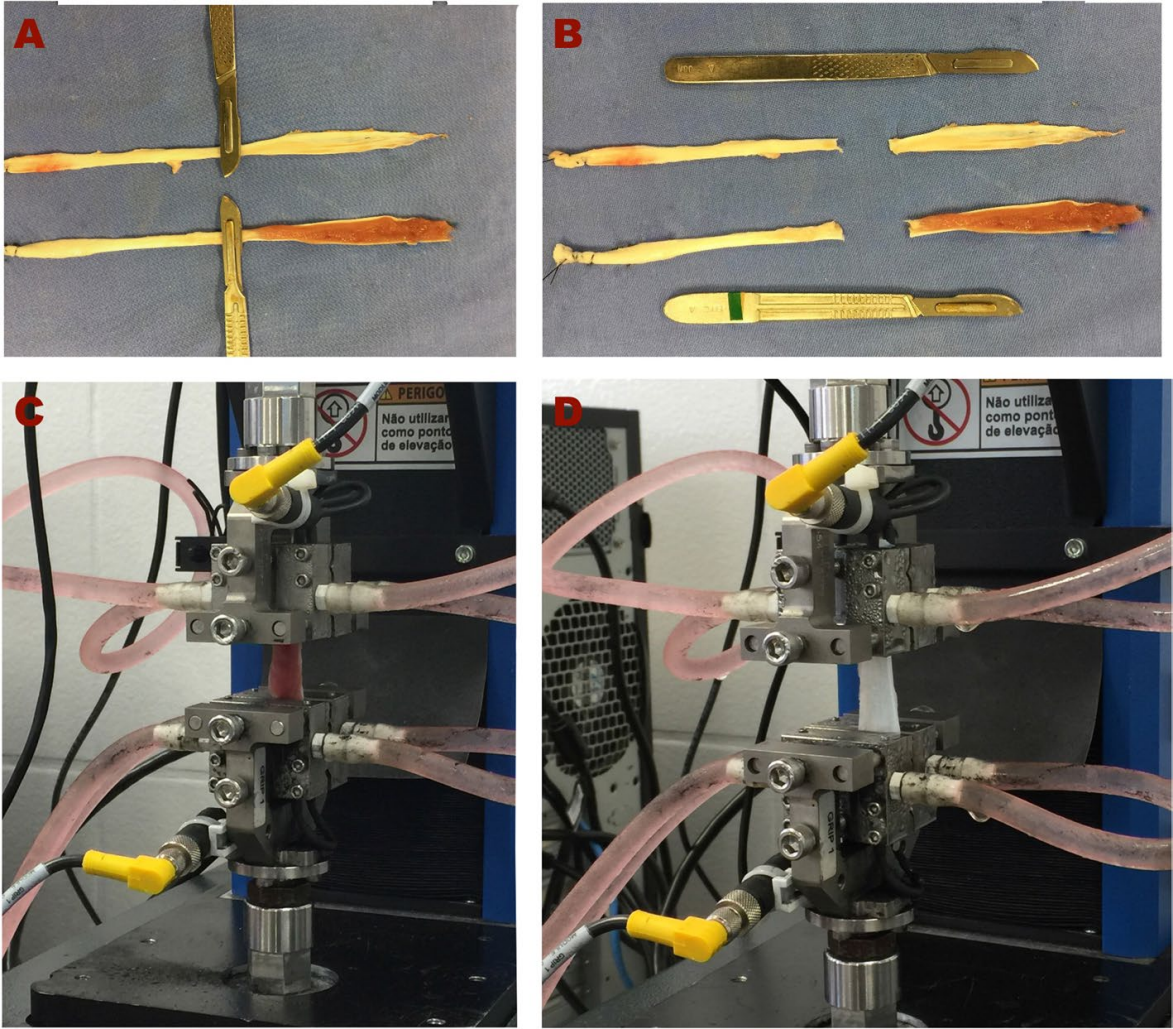
After harvesting the porcine flexor digitorum superficialis muscle tendon (**A**), the musculotendinous tissue was cut for comparison of both tendon tissues under the same conditions (**B**). Each pair of tendons was tested in an Instron TestMaster Automation System: the tendon with muscle (**C**) and the tendon without muscle (**D**). The distance between the tendon fixation points was 30 mm. The cryoclamp (pink liquid inside the hoses) was used to reduce the risk of slippage of the tested structure

The mounting and the mechanical testing were performed on a single limb of graft, using only the portion of tendon with muscle tissue attached in the G1 group (Fig. 4A-B). Fixation of the tendon ends were achieved using the cryoclamps of an Instron TestMaster Automation System, maintaining a 30 mm length. A cryoclamp (a thermally-based soft tissue clamp) was used to avoid soft tissue slippage or rupture at the clamp-tendon interface. These devices are capable of rapid cooling to freeze the specimen and significantly increase the hold force that can be applied without damaging the tissue as shown in Fig. 4C and 4D.

Traction and overload tests were conducted in both groups using an electro-mechanical material testing machine (Instron, model 23-5S, Instron Corp., Canton, MA, USA) with a 500 N force transducer. The tensile testing was carried out under displacement control. The test started with a crosshead velocity of 10 mm/min until reaching 10 N preload. The displacement value was then reset, the crosshead extension rate changed to 20 mm/min, and the tendons were pulled until failure. The variables analyzed and compared in the biomechanical test were as follows: yield load, stiffness, and maximum load.

### Statistical analysis

To evaluate the effect of the muscle stripping process on strength, stiffness, and energy, a two-tailed paired t test with a confidence level of 95% (α = 0.05) was used. An a priori power analysis included the variables strength, resistance, and energy as observed in previous studies and was based on the primary hypothesis that graft harvesting has been associated with a decrease in tissue resistance. It was determined that a sample size of 27 tendons per group would detect a 10% change in resistance index with 80% power and 5% significance.

## Results

The results obtained in the present study showed that there was no difference between groups in relation to gender, weight, and age of the porcine donor (Table 1).

**Table 1:** Demographic data of pigs

	Total	Group 1	Group 2	*p*
Age (weeks)	28.1	28.1	28.1	> 0.05
Weight (kg)	104.9	104.9	104.9	> 0.05
Sex (n)				
Male	40	20	20	> 0.05
Female	42	21	21	

**Fig. 5:**
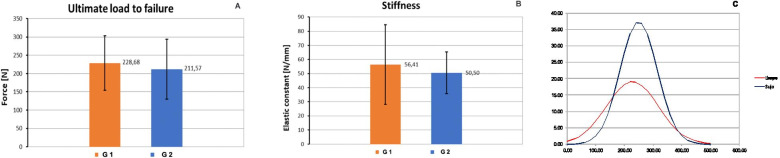
Comparison between the mean force applied in G1 (muscle tissue preservation) and G2 (removal of muscle tissue) for the variables yield load (**A**), tendon stiffness (**B**), and energy up to maximum load (**C**). For all the comparisons, there was no significant difference between groups (*p* = 0.11; 0.1; 0.76, respectively)

**Fig. 6:**
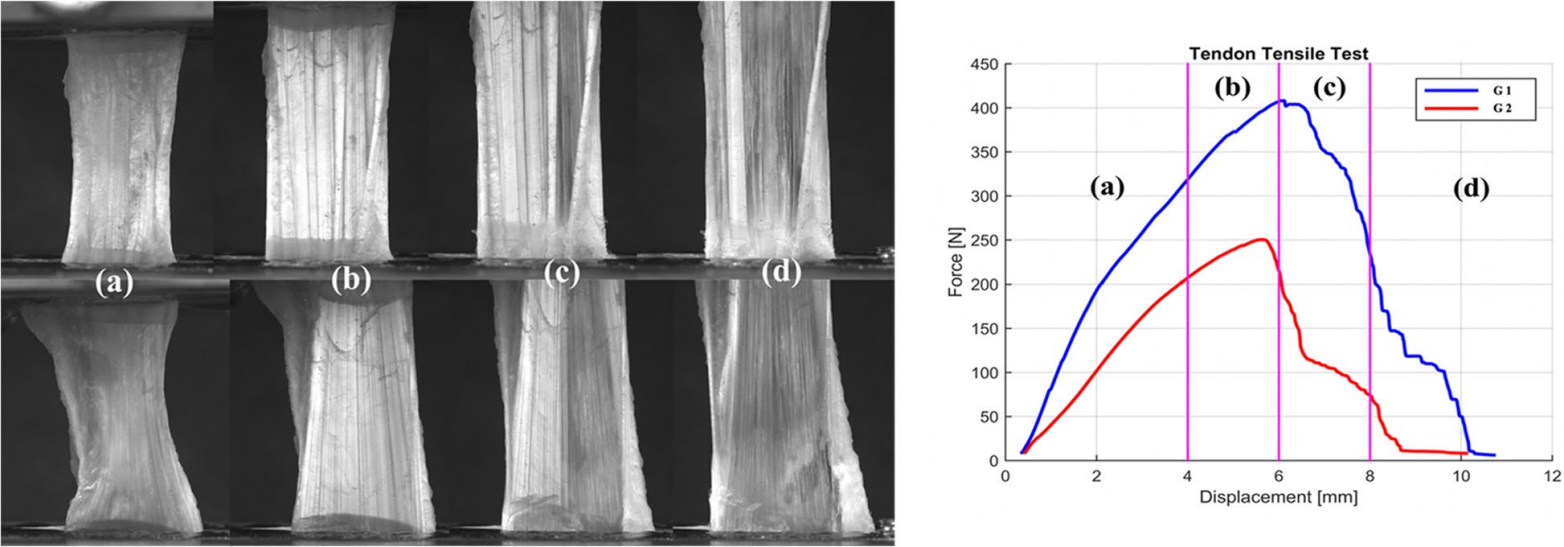
(A) shows comparative images of the tendons in G1 (above) and G2 (below): phase (a) represents the relaxed state of the tendon; phase (b) shows the moment of maximum force sustained by the tendons; phase (c) shows the rupture of the first fibers; and phase (d), tendon failure. (B) illustrates the tensile strength and the deformation of the tendons

The behavior of the porcine grafts during the tests followed the same pattern of stretching, deformation, and failure observed in human grafts subjected to axial strain. There were no significant differences in the comparison between groups for yield load (*p* = 0.105), stiffness (*p* = 0.097), and maximum load (*p* = 0.761), as outlined in Figs. 5 and 6.

## Discussion

The most important finding of the present study was that the preservation of muscle tissue in the harvested porcine flexor digitorum superficialis tendon did not have negative effects on its biomechanical performance when compared to tendon that had its musculature removed. This is in contrast to Sun et al., which concluded that excessive amounts of retained skeletal muscle weaken tendon graft's strength in the setting of ACL reconstruction [28].

In a clinical study, Funchal et al. reported that the use of the knee flexor tendon autograft with the preservation of adjacent muscle tissue demonstrated biological and regenerative potential in patients who underwent ACLR [9]. They showed increased graft size as well as favorable Tegner activity scale and Lysholm scores a minimum of two years postoperatively. Further, the study demonstrated a favorable histological characterization during the healing process. Ćuti et al., presented data supporting the capacity of muscle-derived cells to differentiate into tendon tissue [5], and other studies have also demonstrated how tendon muscle remnants improved ACL graft healing [10] Sun et al. also concluded that muscle left on tendon autografts promoted intra-articular healing and remodeling of the graft [28].

Given the established volumetric increase in final graft diameter with muscle preservation, the need for a biomechanical evaluation of the tendon structure with or without the preservation of muscle tissue was necessary [9]. In the present study, the maximum load, stiffness, and the yield load required for tendon failure were evaluated in a porcine model. The preservation of muscle tissue on the grafts tested in this study did not compromise its biomechanical performance. This demonstrates that, in addition to the cellular level advantages, graft muscle preservation does not compromise biomechanical properties when compared to a purely tendon autograft.

Although the biomechanical testing was performed in a porcine model, the flexor digitorum superficialis was used which has comparable anatomy to the hamstring tendons in the human knee. Previous studies have already shown that the porcine flexor digitorum superficialis tendons are considered comparable to human hamstring tendons in relation to the anatomical and histological characteristics [12, 16]. Besides, according to Woo et al., their biomechanical properties demonstrate a rate of deformity similar to lower limbs human tendons [33].

Thus, this biomechanical analysis of the musculotendinous tissue of a pig represents what could be expected in human tissue. The present study also demonstrated that the porcine tendon properties displayed during testing were similar to the pattern of stretching, deformation, and failure observed in human tendons under the effect of axial strain [7, 20].

The effect of removal of muscle tissue from tendon grafts on tendon strength was previously evaluated by Sun et al. Their results were similar to that of the present study, despite the use of different methodology [28]. The time zero biomechanical properties of the grafts are only part of the equation, and there are deleterious histological effects on tendon grafts subjected to muscle stripping.

The weakening of the graft caused by muscle removal starts during surgery at the time of graft preparation and may have deleterious effects on the "ligamentization" process and ligament remodeling. Okazaki et al., demonstrated that muscle stripping of the hamstring tendons caused histological alterations and damage to type I collagen. The density of type I collagen fibers decreased with increasing number of strips necessary to remove musculature [17].

The biological benefits and robust biomechanical profile of grafts with preserved muscle attachments suggest that their use is a reasonable practice especially given the traumatic effect of muscle stripping on a graft’s cellular properties.

### Study limitations

The present study has some limitations. First, porcine tendons were used instead of human tendons. This choice, however, was made due to their immediate availability and low cost. Second, the single strand tendon used did not represent the graft diameter in a real clinical situation. Finally, the time zero nature of biomechanical studies was unable to evaluate the potential of improved healing in grafts with preserved muscle. Improved healing of grafts with preserved muscle may be clinically relevant as a way to improve the healing of ACL reconstruction in humans.

## Conclusion

In this porcine model biomechanical study, using autograft tendon with preserved muscle showed no statistically significant differences for yield load, stiffness, or maximum load compared to autograft tendon without preserved muscle. The preservation of muscle on the autograft tendon did not compromise the mechanical properties of the autograft.
